# Supplementary material for: Circulating Tumour DNA (ctDNA) as a Predictor of Clinical Outcome in Non-Small Cell Lung Cancer Undergoing Targeted Therapies: A Systematic Review and Meta-Analysis
Source: Cancers (Basel). 2023 Apr 23;15(9):2425. doi: 10.3390/cancers15092425 (PMC10177293; doi:10.3390/cancers15092425)
Supplement: Supplementary file 1 [file cancers-15-02425-s001.zip › cancers-2320601-supplementary.pptx]

## Slide 1
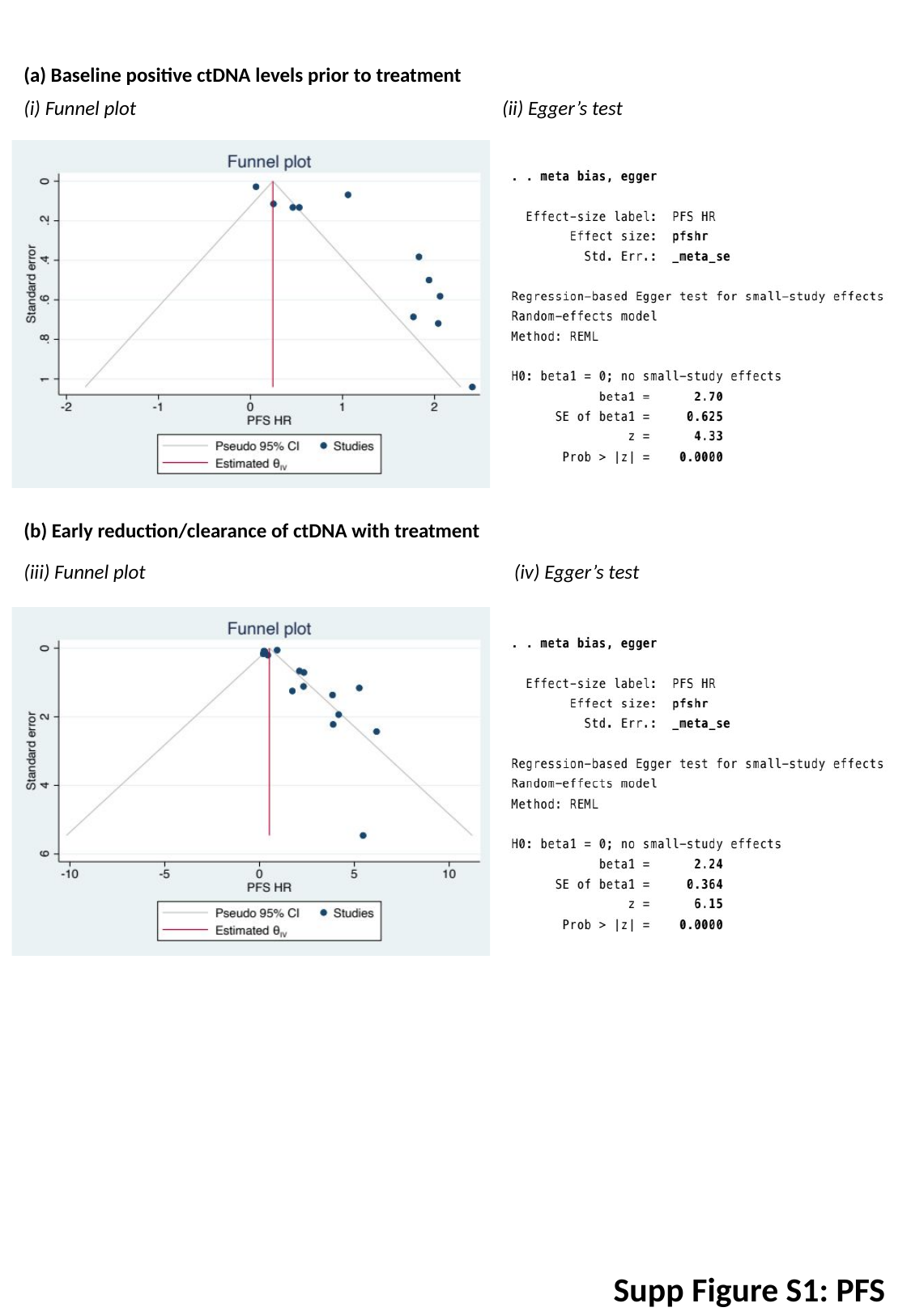

(a) Baseline positive ctDNA levels prior to treatment
(i) Funnel plot
(ii) Egger’s test
(b) Early reduction/clearance of ctDNA with treatment
(iii) Funnel plot
(iv) Egger’s test
Supp Figure S1: PFS

## Slide 2
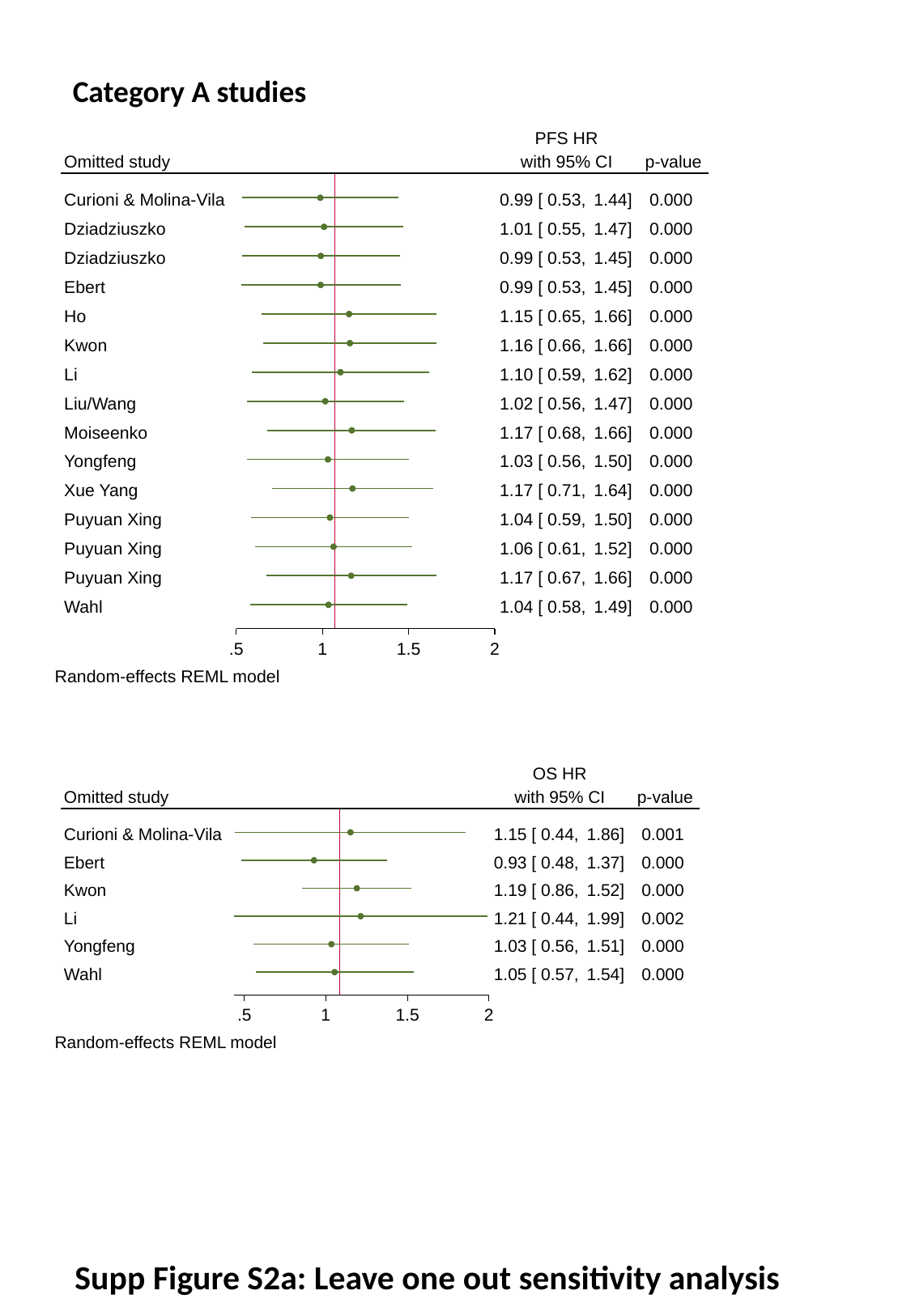

Category A studies
Supp Figure S2a: Leave one out sensitivity analysis

## Slide 3
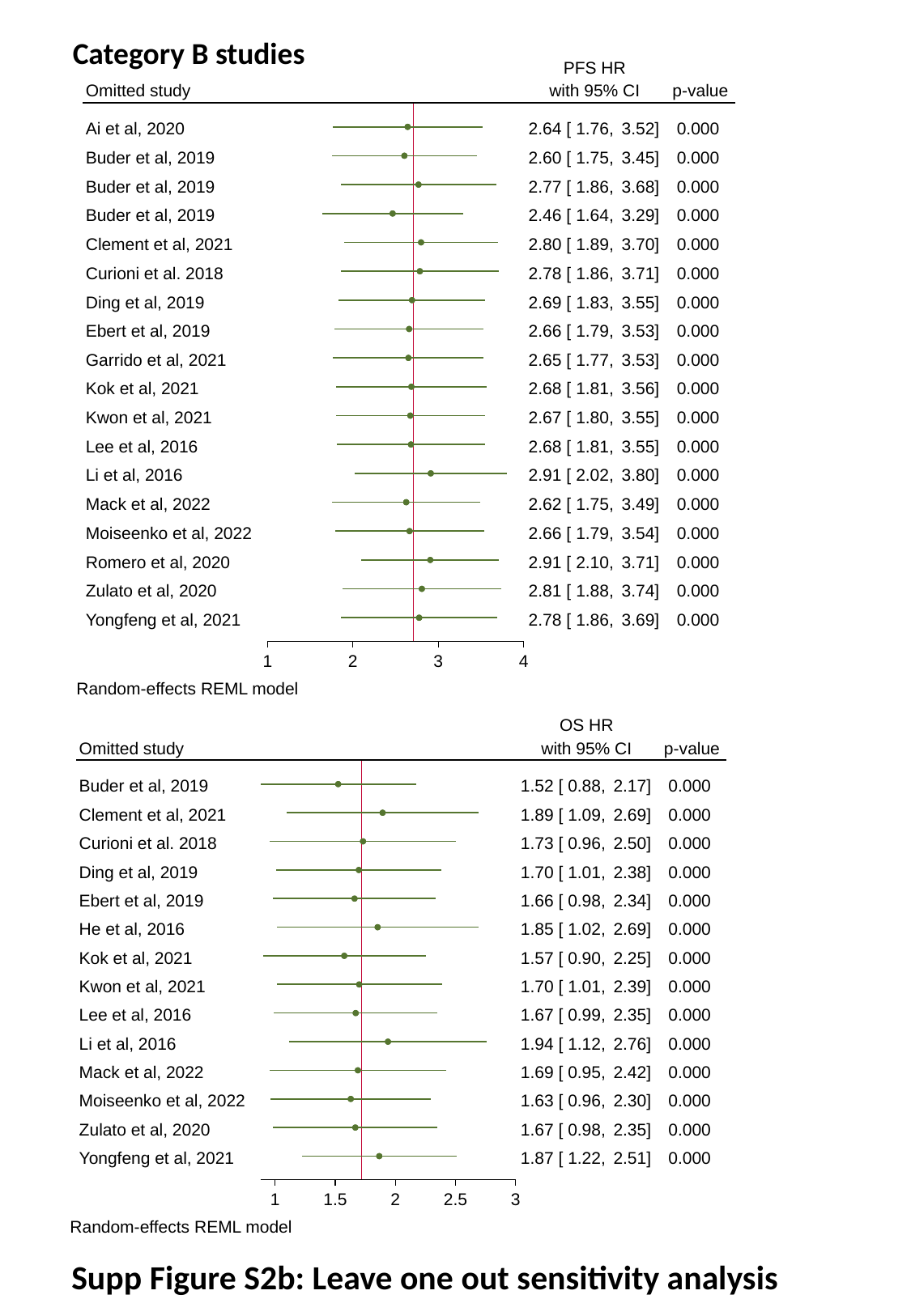

Category B studies
Supp Figure S2b: Leave one out sensitivity analysis

## Slide 4
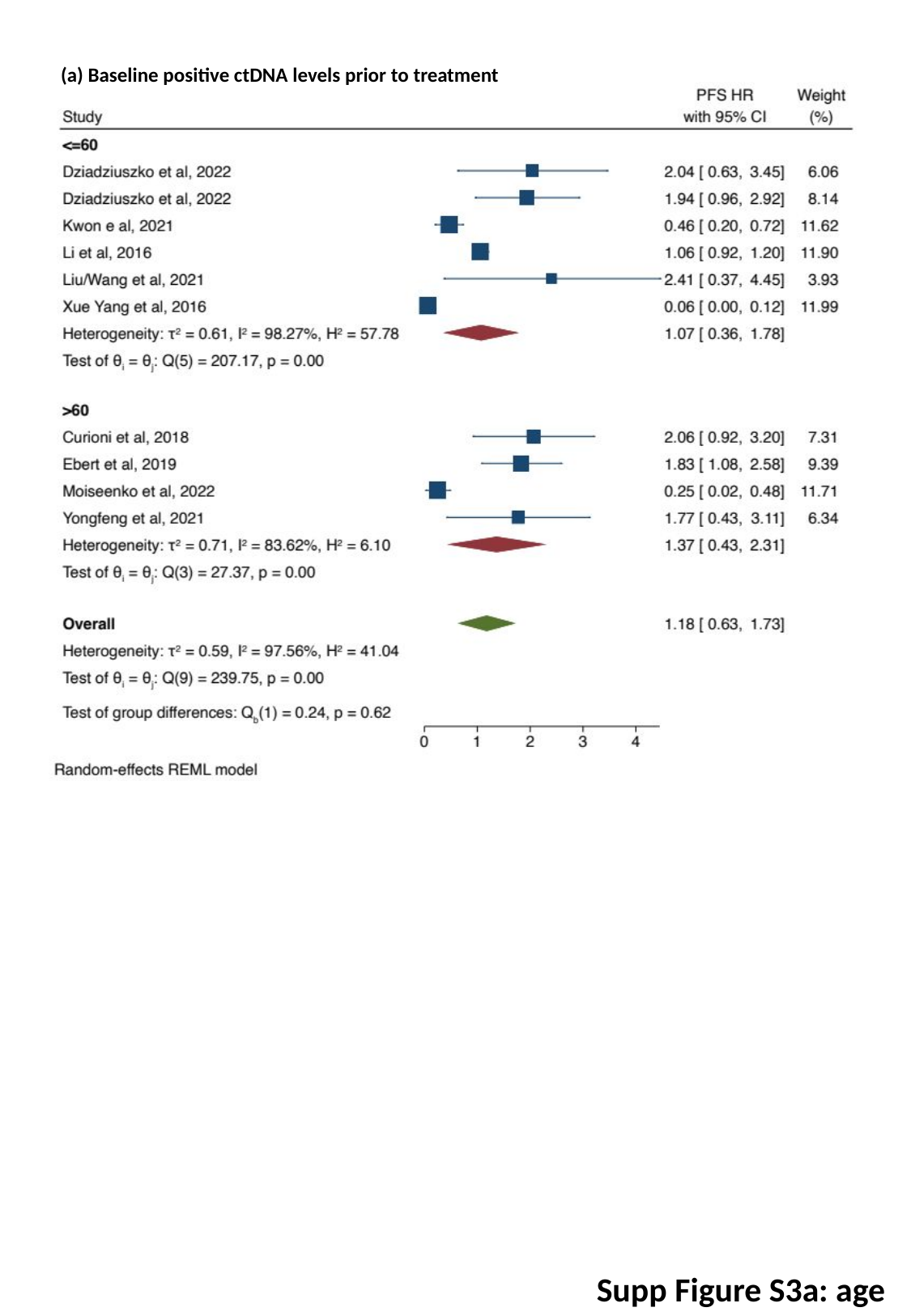

(a) Baseline positive ctDNA levels prior to treatment
Supp Figure S3a: age

## Slide 5
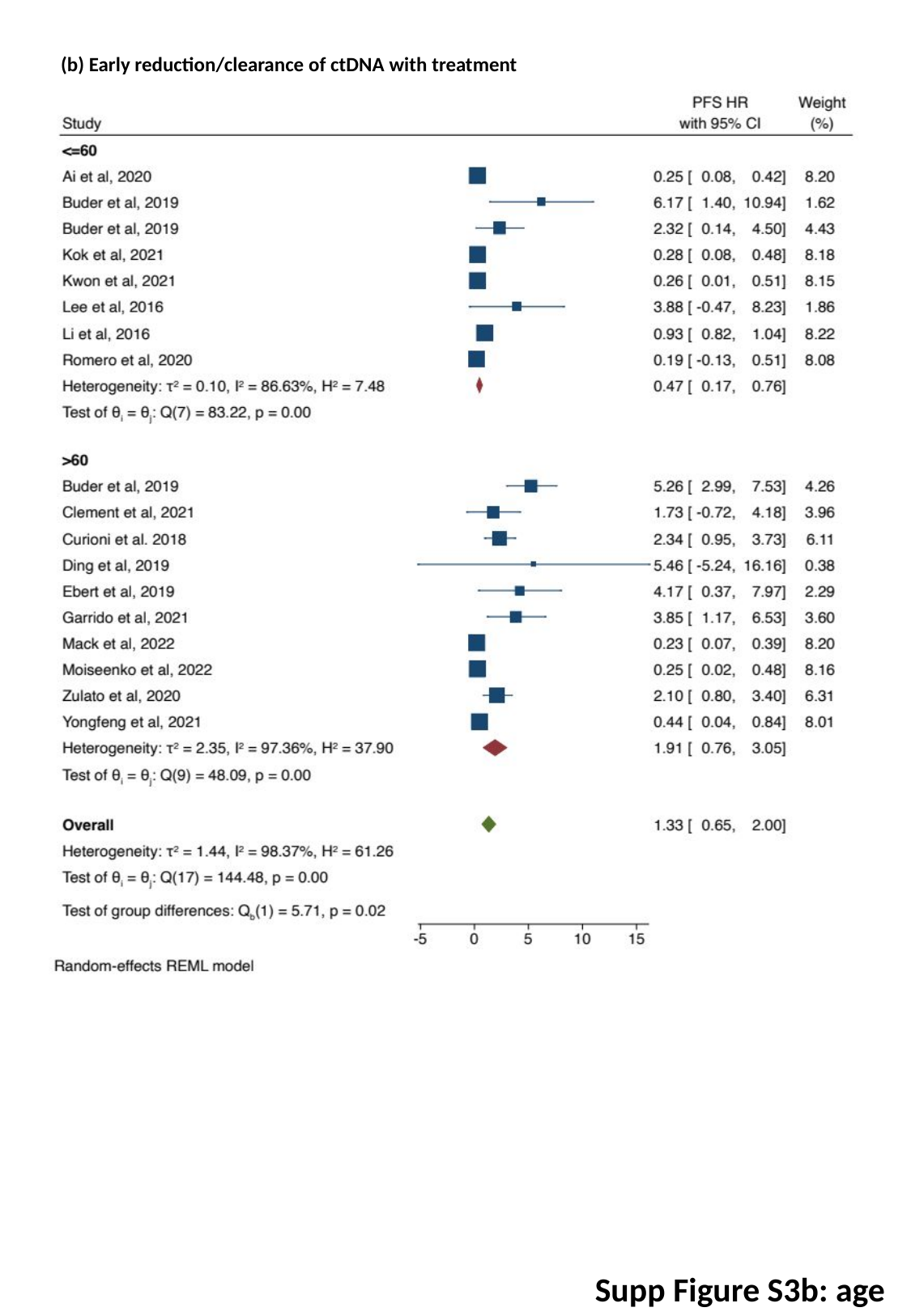

(b) Early reduction/clearance of ctDNA with treatment
Supp Figure S3b: age

## Slide 6
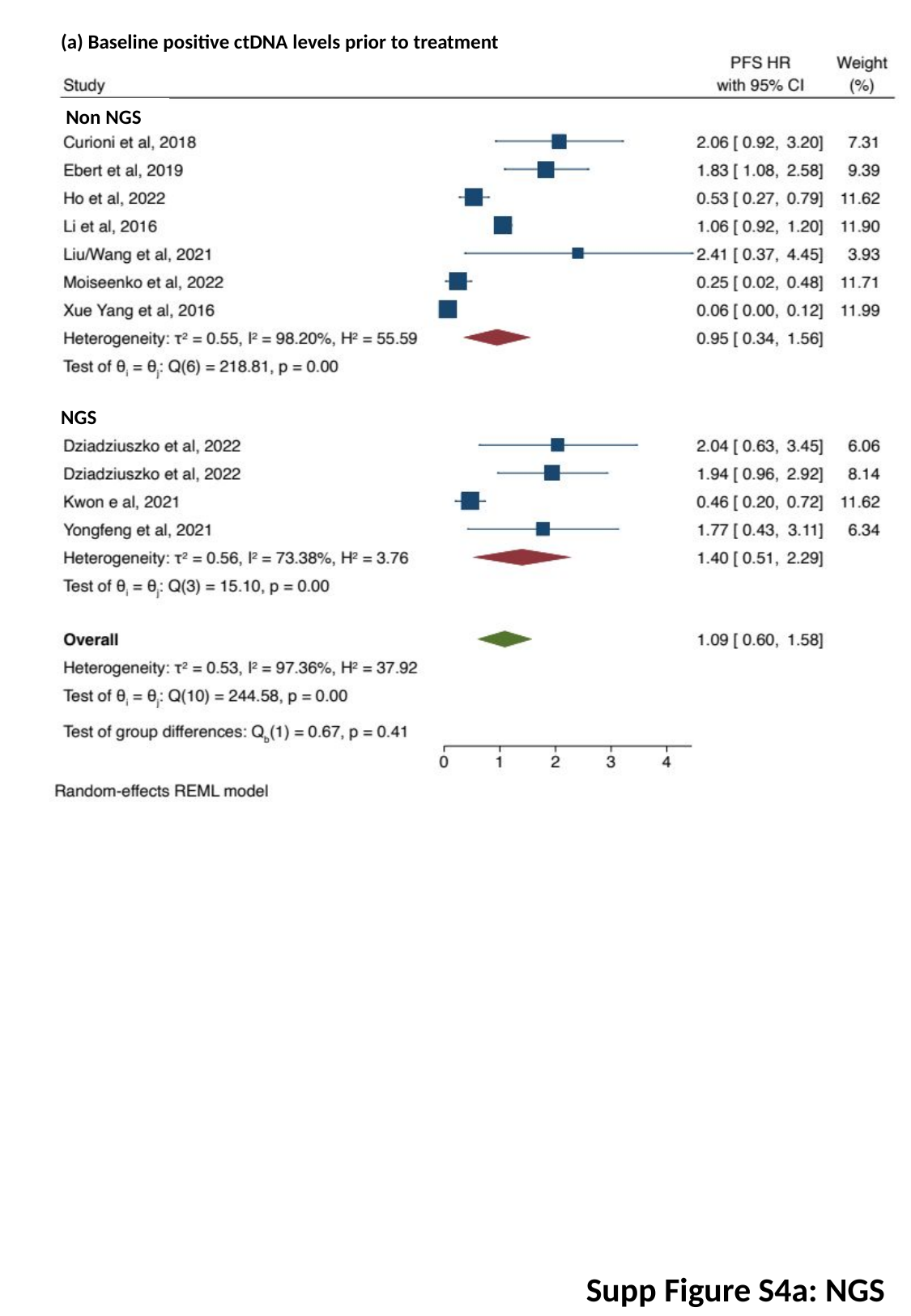

(a) Baseline positive ctDNA levels prior to treatment
Non NGS
NGS
Supp Figure S4a: NGS

## Slide 7
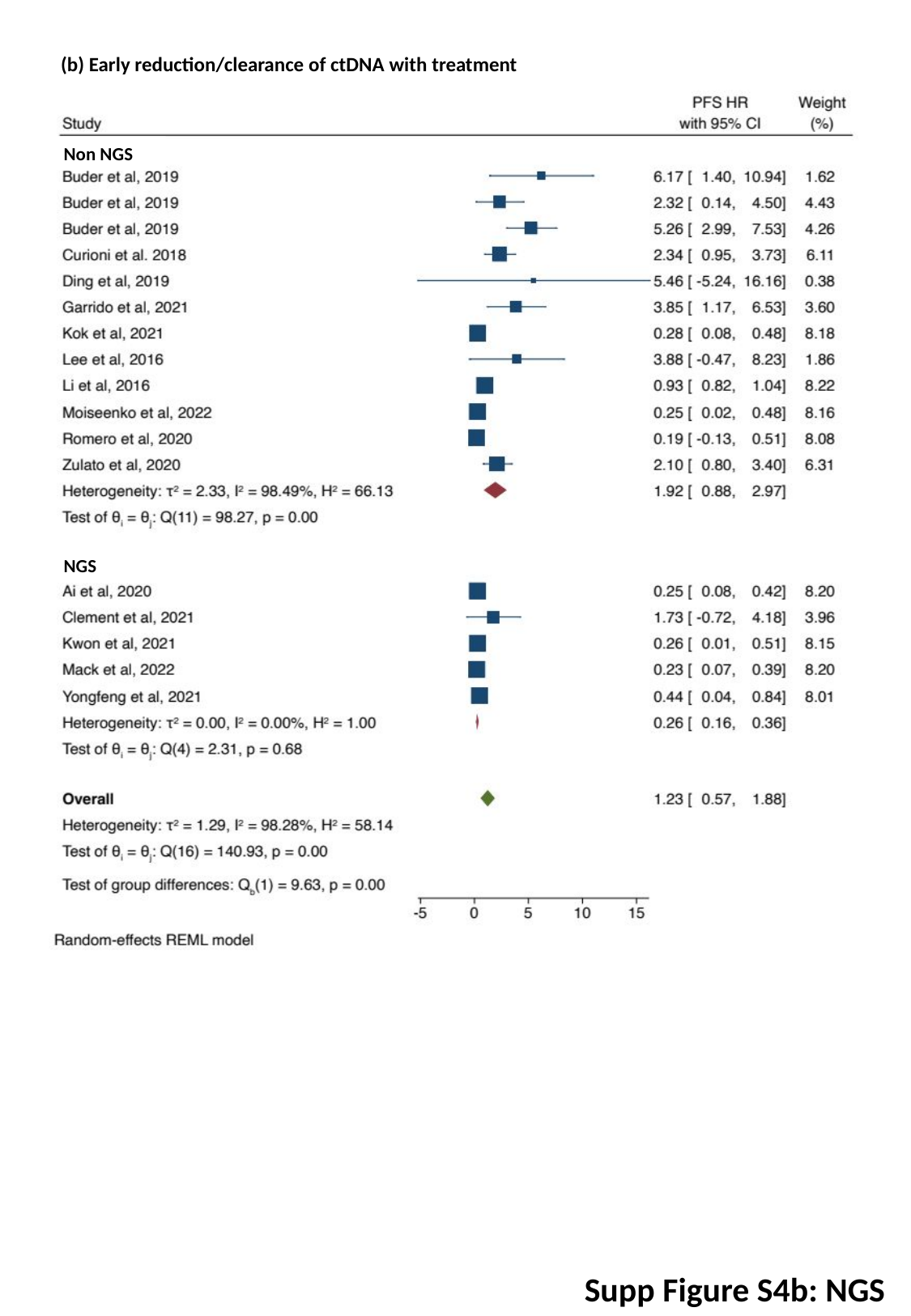

(b) Early reduction/clearance of ctDNA with treatment
Non NGS
NGS
Supp Figure S4b: NGS

## Slide 8
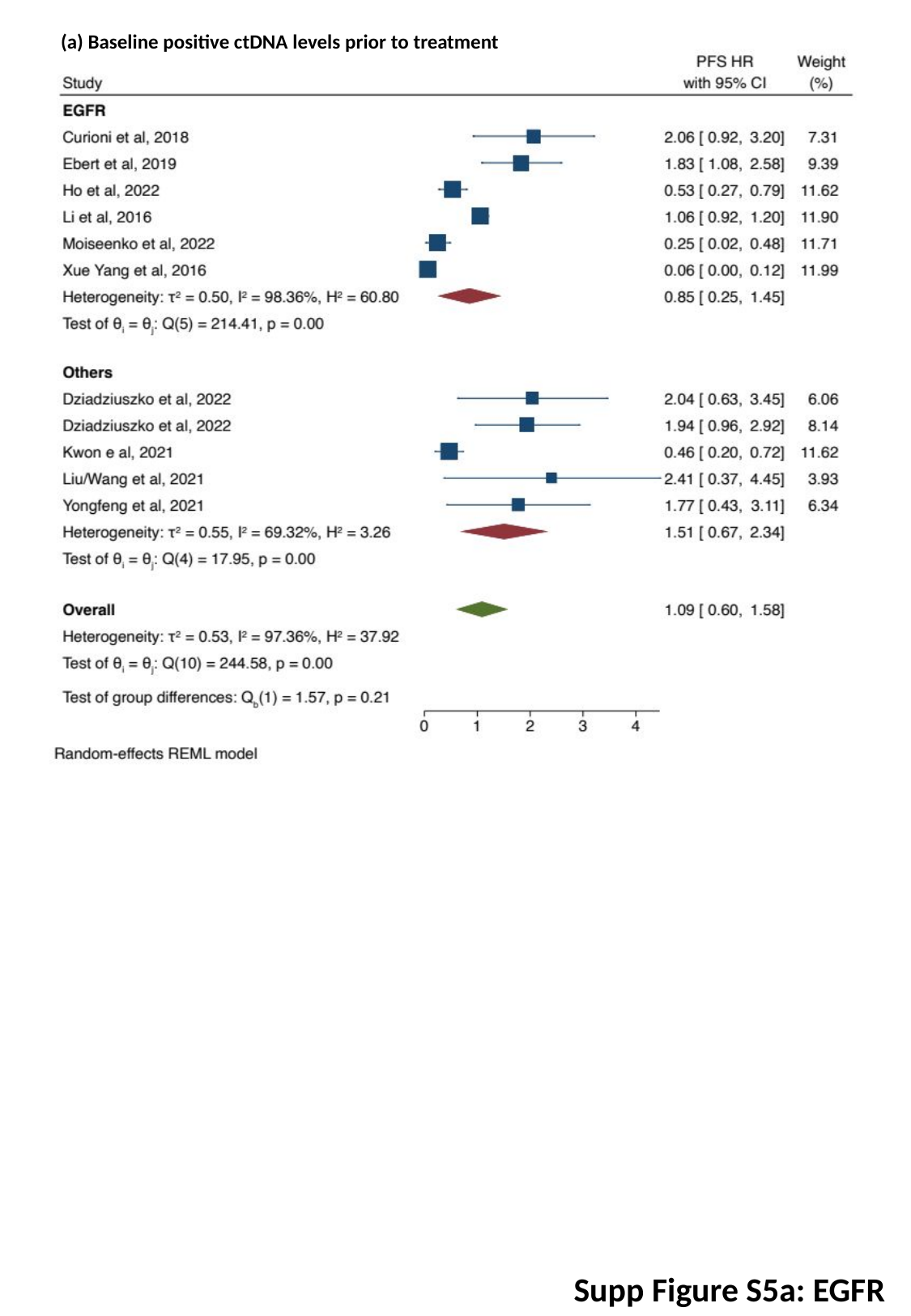

(a) Baseline positive ctDNA levels prior to treatment
Supp Figure S5a: EGFR

## Slide 9
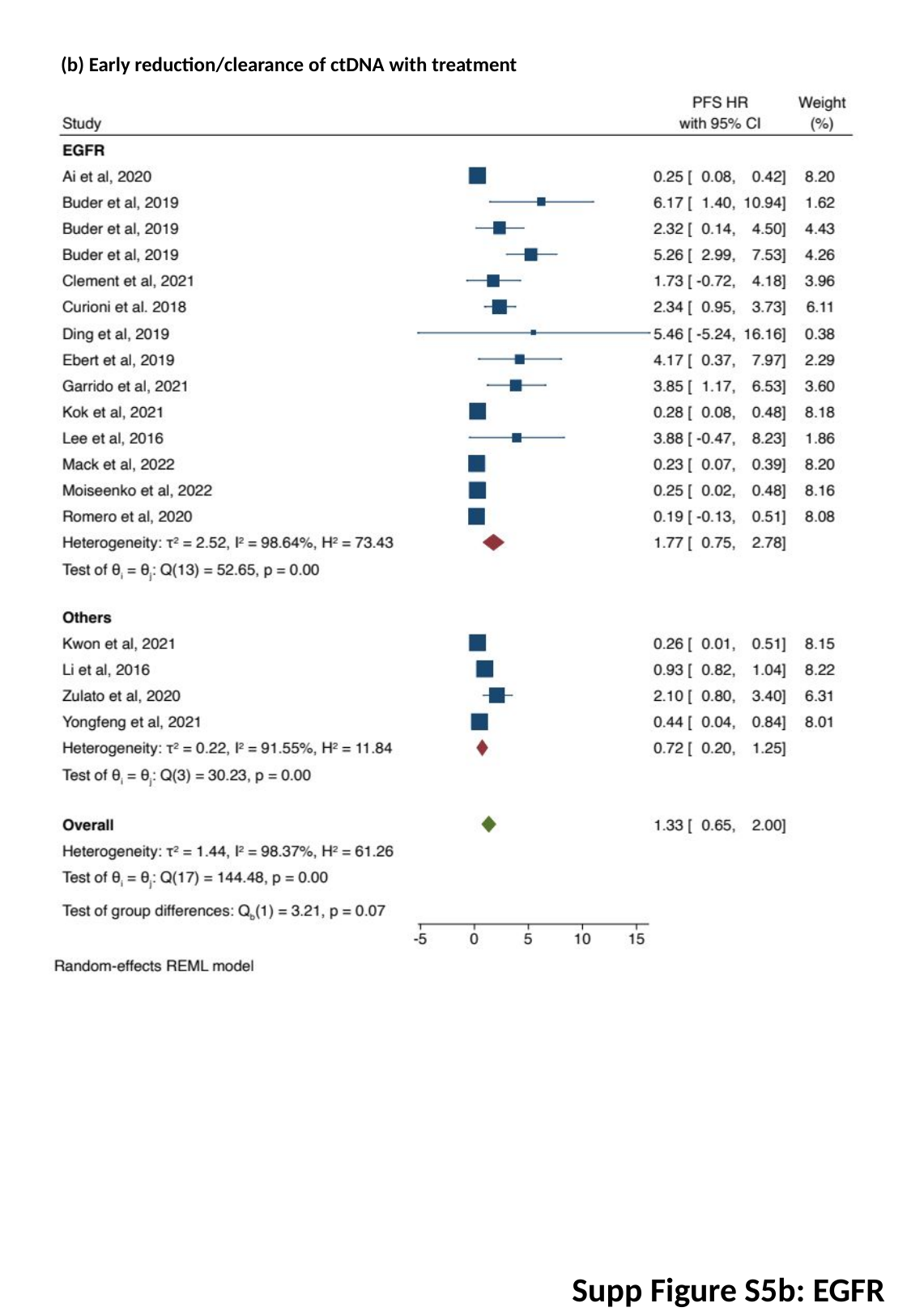

(b) Early reduction/clearance of ctDNA with treatment
Supp Figure S5b: EGFR

## Slide 10
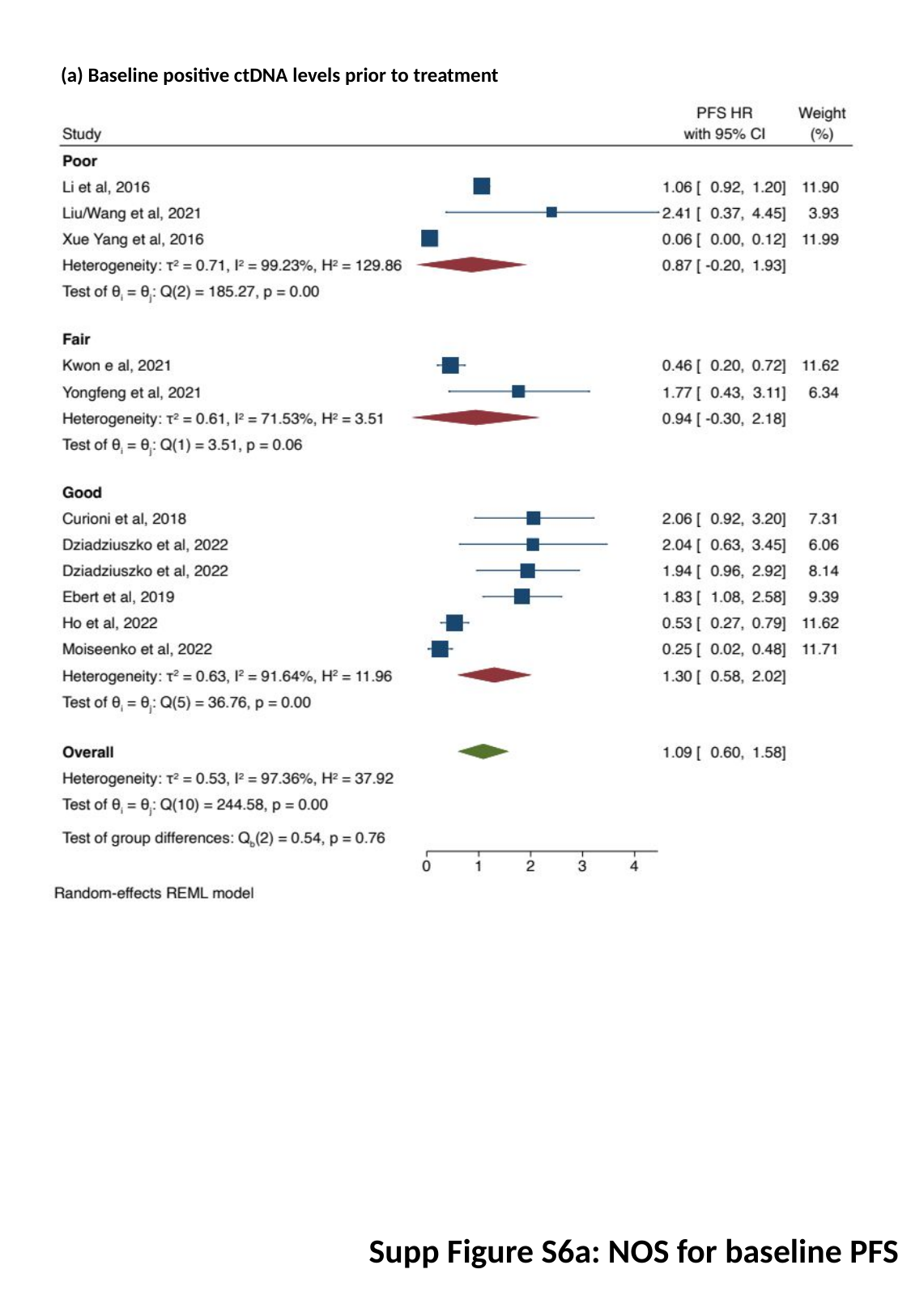

(a) Baseline positive ctDNA levels prior to treatment
Supp Figure S6a: NOS for baseline PFS

## Slide 11
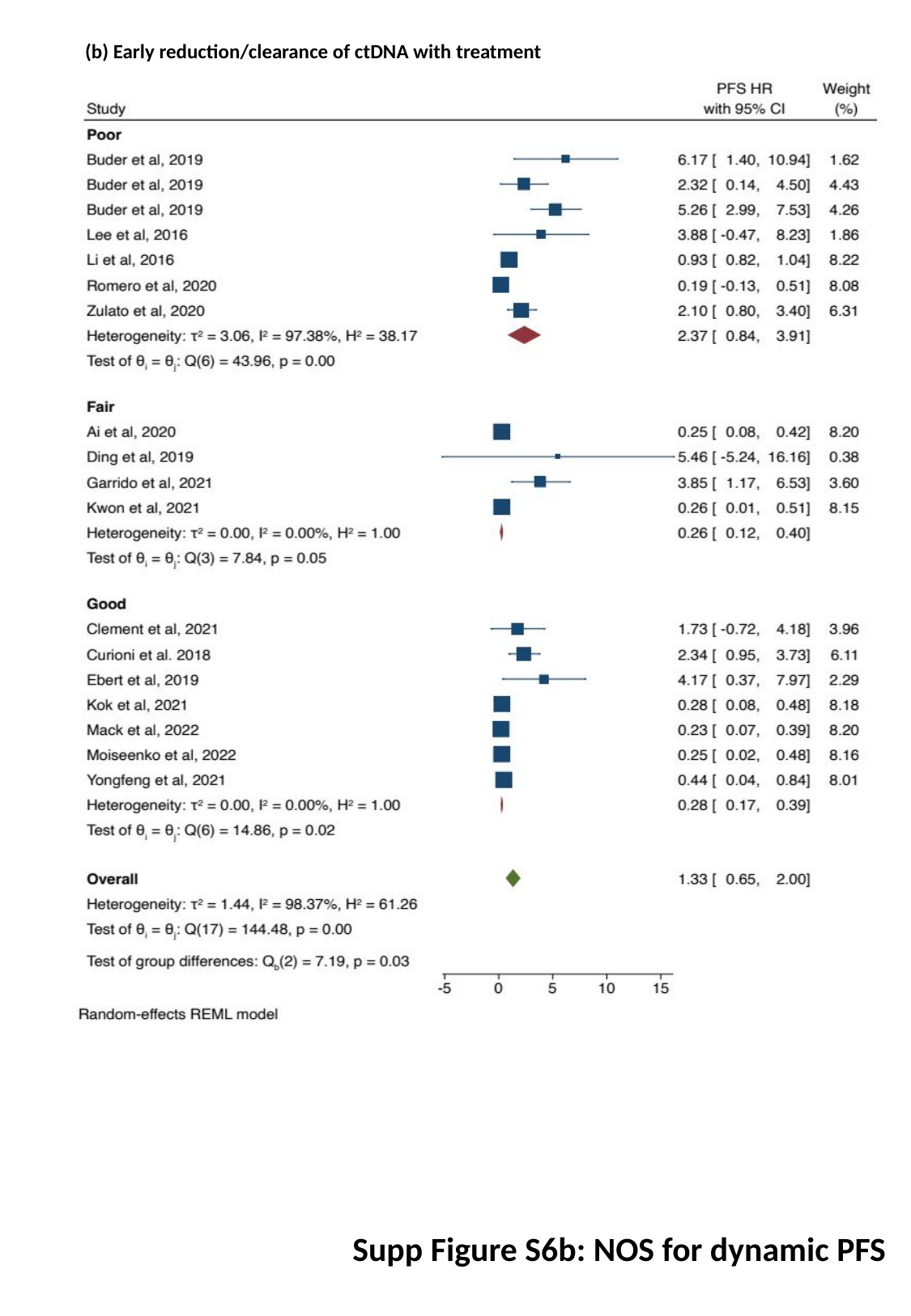

(b) Early reduction/clearance of ctDNA with treatment
Supp Figure S6b: NOS for dynamic PFS

## Slide 12
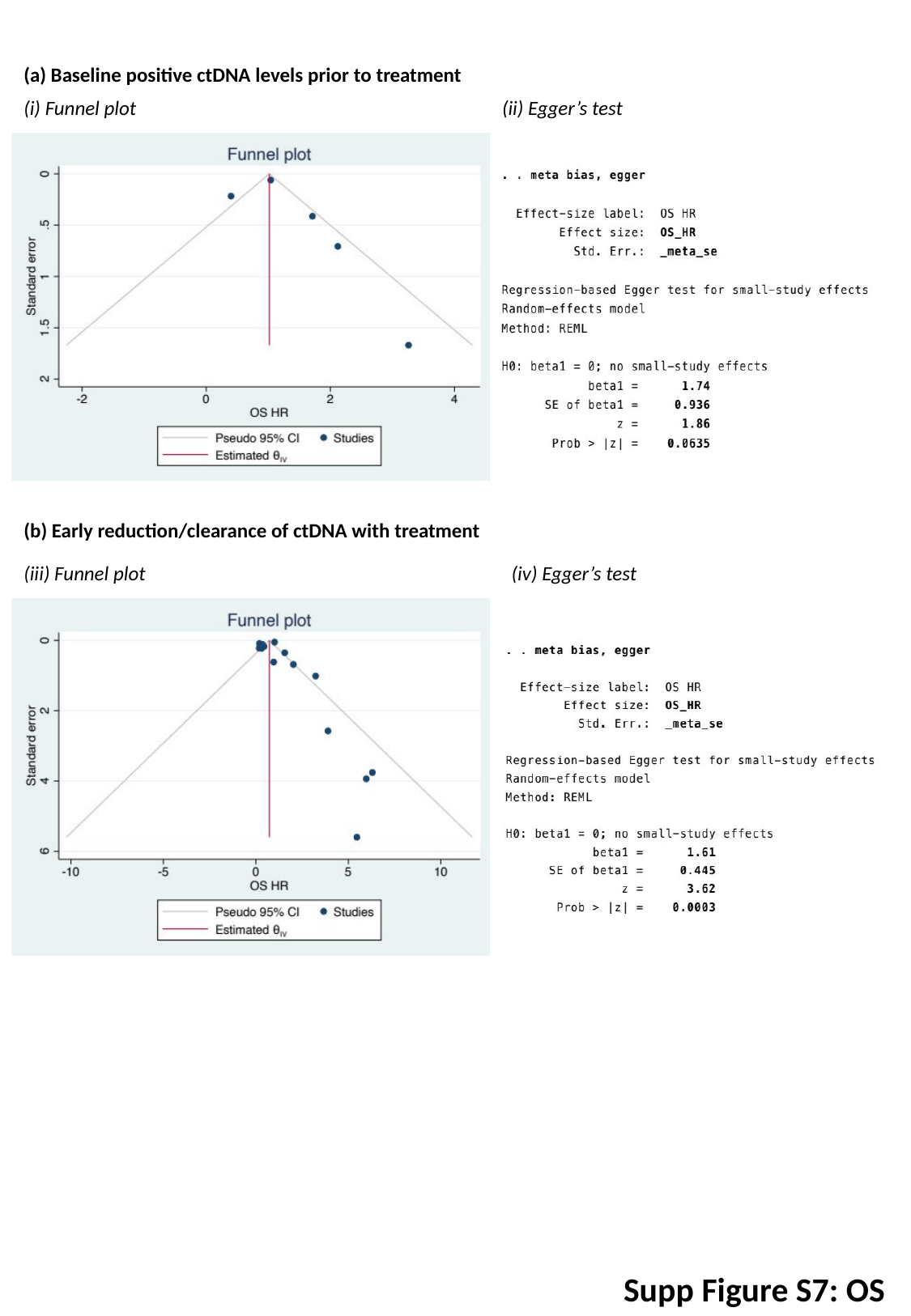

(a) Baseline positive ctDNA levels prior to treatment
(i) Funnel plot
(ii) Egger’s test
(b) Early reduction/clearance of ctDNA with treatment
(iv) Egger’s test
(iii) Funnel plot
Supp Figure S7: OS

## Slide 13
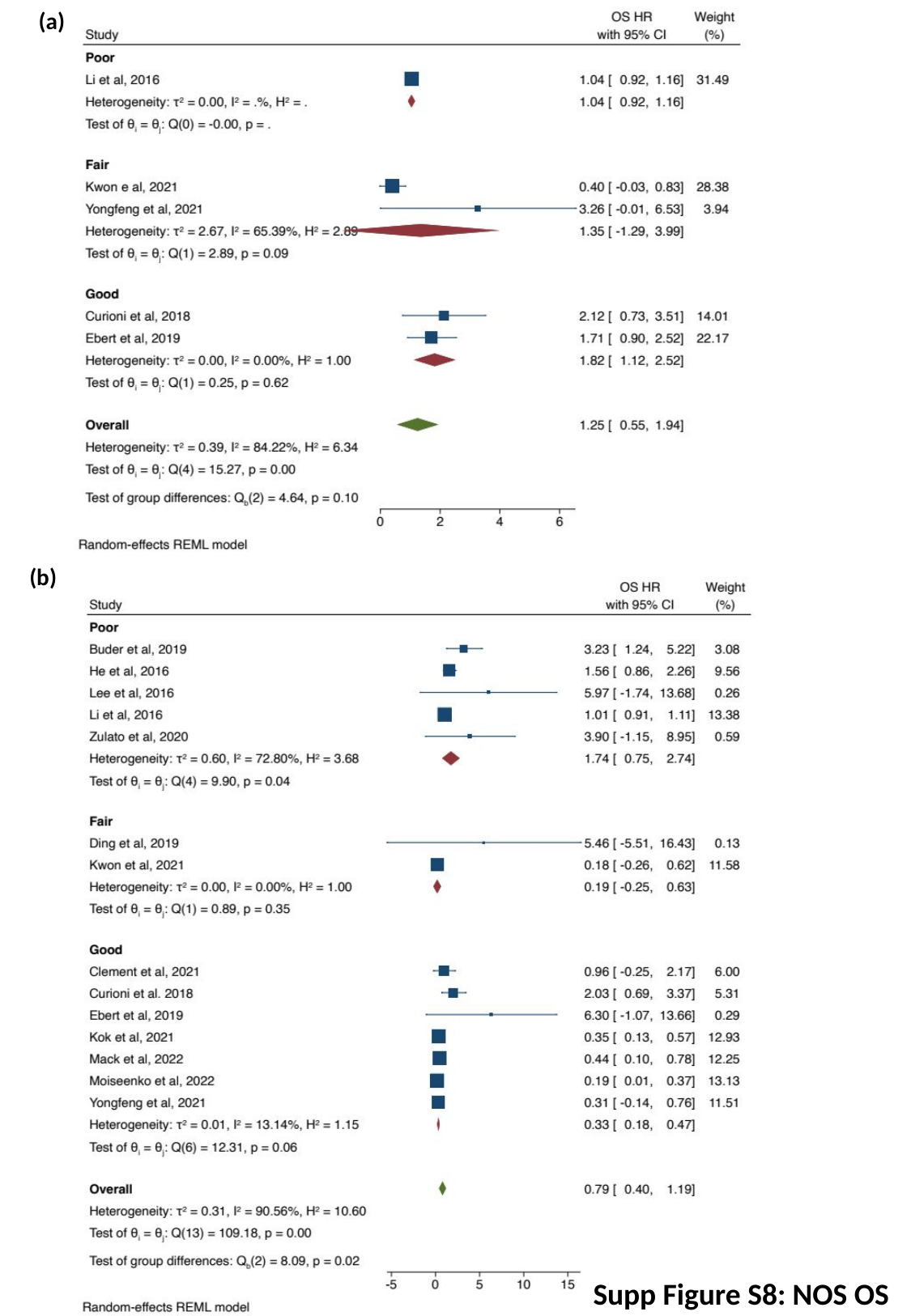

(a)
(b)
Supp Figure S8: NOS OS

## Slide 14
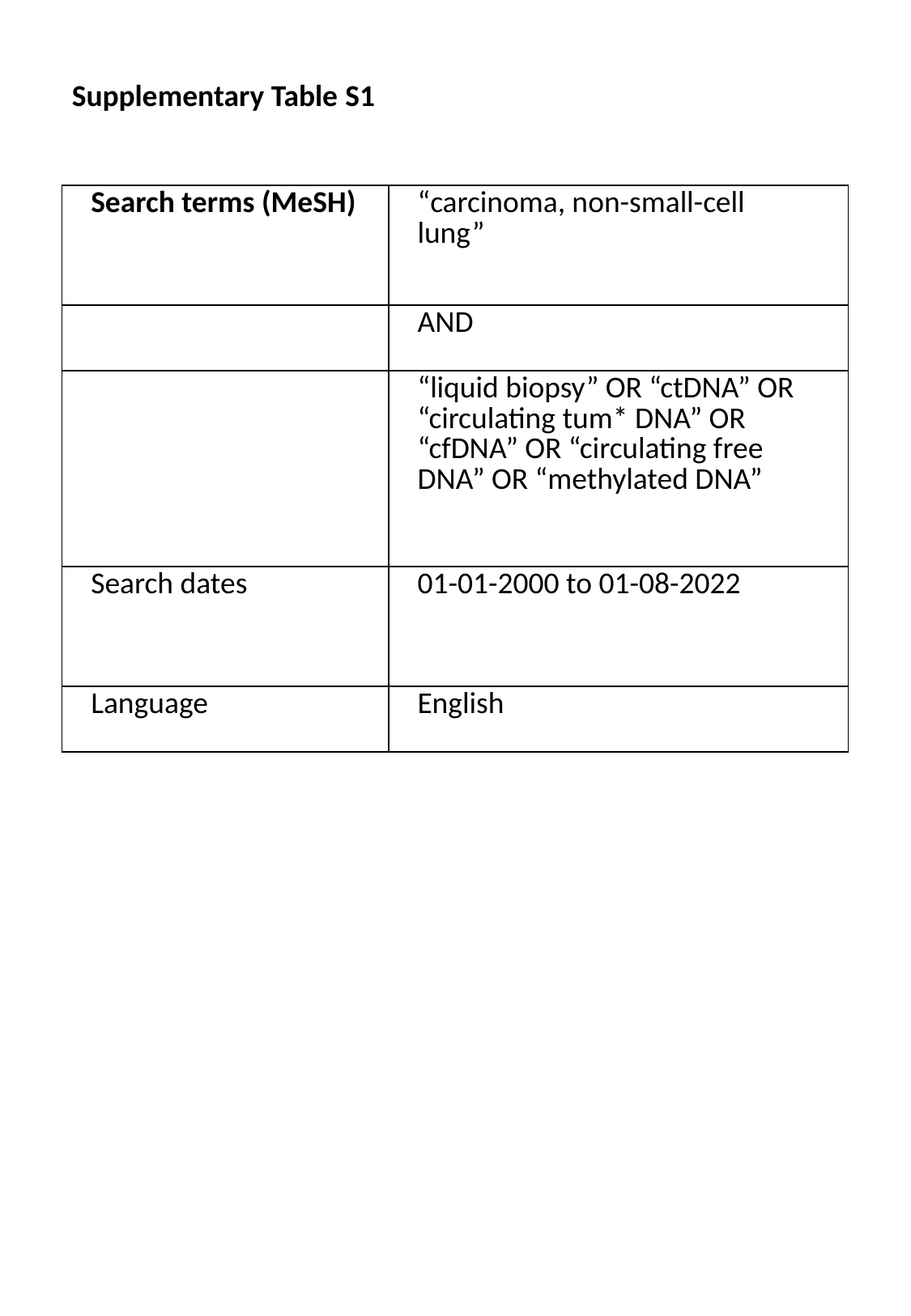

Supplementary Table S1
| Search terms (MeSH) | “carcinoma, non-small-cell lung” |
| --- | --- |
| | AND |
| | “liquid biopsy” OR “ctDNA” OR “circulating tum\* DNA” OR “cfDNA” OR “circulating free DNA” OR “methylated DNA” |
| Search dates | 01-01-2000 to 01-08-2022 |
| Language | English |
